# Comparison of Findings between Clinical Examinations and Drug-Induced Sleep Endoscopy in Patients with Obstructive Sleep Apnea Syndrome

**DOI:** 10.3390/ijerph17176041

**Published:** 2020-08-19

**Authors:** Huan-Yu Lin, Yi-Chih Lin, Ying-Shuo Hsu, Liang-Chun Shih, Tyler Nelson, Wen-Dien Chang, Yung-An Tsou

**Affiliations:** 1 Department of Otorhinolaryngology, China Medical University Hospital, Taichung 40402, Taiwan; cmupuma@gmail.com (H.-Y.L.); entdrshih7111@gmail.com (L.-C.S.); 2 Department of Otolaryngology, Taipei Medical University Shuang Ho Hospital, Taipei 23561, Taiwan; yeast1123@hotmail.com; 3 Department of Otolaryngology, Shin Kong Wu Ho-Su Memorial Hospital, Taipei 11101, Taiwan; sulisky@gmail.com; 4 School of Medicine, Fu-Jen Catholic University, Taipei 242062, Taiwan; 5 Graduate Institute of Biomedical Sciences, China Medical University, Taichung 40402, Taiwan; 6 Department of Biology, Lamar University, Beaumont, TX 77710, USA; tnelson@lamar.edu; 7 Department of Sport Performance, National Taiwan University of Sport, Taichung 40404, Taiwan; 8 Department of Otolaryngology-Head and Neck Surgery, China Medical University Hospital, Taichung 40402, Taiwan; 9 Department of Audiology and Speech Pathology, Asia University, Taichung 41354, Taiwan

**Keywords:** drug-induced sleep endoscopy, Brodsky classification, Friedman’s staging system, obstructive sleep apnea syndrome

## Abstract

The Velum, Oropharynx, Tongue base and Epiglottis (VOTE) classification on drug-induced sleep endoscopy (DISE) is used widely for obstructive sleep apnea (OSA) syndrome, though research into comparative physical examinations with VOTE on DISE is still limited. The aim of this study was to evaluate the relationship between the findings of physical examinations and DISE in patients with OSA. Fifty-five patients with OSA were enrolled in this retrospective study. All of the patients received clinical explorations including a Brodsky classification, a modified Mallampati score (MMS), a modified Friedman’s staging system, and a Muller’s test. Drug-induced sleep endoscopy was further evaluated in the operating room. There were significant relationships between Brodsky classification, modified Friedman’s staging system, Muller’s test and oropharynx collapse during DISE (*p* < 0.05). Brodsky classification, MMS, modified Friedman’s staging system and retropalatal lateral-to-lateral (L–L) collapse of Muller’s test were significantly correlated with VOTE count (*p* < 0.05). The concordance between VOTE under DISE and Brodsky classification or modified Friedman’s staging system was moderate. In contrast, the concordance between VOTE under DISE and MMS or Muller’s test was slight. The study revealed that Brodsky classification and Friedman staging had a significant relationship with DISE on the velum and oropharynx, but the level of tongue base is uncertain between DISE and MMS. Correlation of awake evaluation of tongue base is still not correlated to the DISE findings. Pre-treatment evaluation of DISE is still warranted.

## 1. Introduction

Obstructive sleep apnea (OSA) syndrome is characterized by a repetitive upper airway collapse during sleep, narrowing the pathway for airflow and causing periods with hypopnea and apnea with decreased blood oxygen levels [1]. Polysomnography (PSG), first described in 1965 by Gastaut, is utilized to diagnose the severity of OSA [2]. Fundamentally, continuous positive airway pressure (CPAP) therapy remains the gold standard for the conservative treatment of OSA. However, surgery may be indicated to improve compliance and outcomes in patients with poor tolerance to CPAP [3,4]. PSG is a gold standard to assess sleep diseases, but it also has some disadvantages, such as sleep testing and data recording all night, performing in an unfamiliar environment, and affecting normal sleep due to limited channel monitoring. Croft et al. first tried to use endoscopy for upper airway under sedation [5], and now drug-induced sleep endoscopy (DISE) is used widely for OSA. DISE could assess the upper airway similar to natural sleep via endoscopic evaluation. It has high validity and reliability for the identification of specific anatomical structures that contribute to obstruction [6,7]. Hence, DISE is another ideal assessment tool for OSA. Type I PSG is the gold standard diagnostic tool for sleep disorders and does not make a topographic diagnosis, whereas DISE is a dynamic study to identify the anatomical site of upper airway collapse in order to improve surgical success.

The level of obstruction is essential for determining correct surgical management of patients with OSA. Conventionally, airway evaluation has relied on physical examination with Brodsky classification, modified Mallampati score (MMS), and modified Friedman’s staging system. Brodsky classification is used for palatine tonsil size evaluation [8]. MMS is used for grading visibility of oropharynx between the palate and tongue based on tongue size and oral cavity; higher MMS refers to a larger tongue size [9]. Modified Friedman’s staging system is used to predict the success of uvulopalatopharyngoplasty [10]. In addition, pre-treatment Muller’s test is often applied for physical examination on OSA patients in an awake condition to check the collapsibility of velum, oropharynx, tongue base, and even epiglottis in a negative pressure by closing the nose and oral cavity and trying to breathe in simultaneously [11]. However, all of the above-mentioned evaluation is performed in an awake state. In the past two decades, DISE has been used as a method for the three levels of upper airway evaluation during pharmacologically induced sleep [12]. The Velum, Oropharynx, Tongue base, and Epiglottis (VOTE) classification on DISE was used to score the level and degree of upper airway narrowing [13]. The research into correlation between physical examinations, such as Brodsky classification, MMS, modified Friedman’s staging system and Muller’s test, and VOTE on DISE was still inconclusive. Therefore, the aim of this study was to investigate the relationship between the physical examinations and DISE.

## 2. Methods

### 2.1. Study Design

This is a retrospective study. The charts of fifty five patients with OSA were enrolled for review from January 2016 to July 2018 in China Medical University Hospital. This study for waiver of informed consent was approved by the local ethics committee of China Medical University Hospital(No. CMUH103-REC1-078). The inclusion criteria for this study were: (1) adult (age older than 18), (2) apnea-hypopnea index (AHI) > 5 per hour by PSG, and (3) having the records of physical examinations, such as Brodsky classification, MMS, modified Friedman’s staging system and Muller’s test. The exclusion criteria were: (1) previous sleep surgery history, (2) contraindication for anaesthesia. Overnight PSG was performed at a sleep center, which equates to a whole night of PSG, the number of channels included electrocardiogram, electrooculography, electromyography, O2 oximeter, flowmeter, and nasal–oral thermocouple and nasal pressure cannula. The AHI was clarified as events per hour, and evaluated by type I PSG, together with the definitions of apneas and hypopneas under the American Academy of Sleep Medicine (AASM) scoring manual v2.5 edited by an “accepted” scoring rule. The incomplete awake diagnostic tests were excluded as well. The medical records of the patients were reviewed. Basic data, including age and body mass index (BMI), were recorded. Physical examinations including Brodsky classification, MMS, modified Friedman’s staging system and Muller’s test were performed by a senior sleep surgeon with a qualified sleep fellow. The DISE was evaluated via VOTE under total intravenous anesthesia (TIVA) in target control infusion (TCI) mode used with propofol as the sedating agent, administered by a senior sleep surgeon together with a senior anesthesiologist in the operation room. Each of the clinical awakening clinical physical examinations was surveyed and correlations with DISE was assessed by VOTE.

### 2.2. Assessments of Physical Examination

#### 2.2.1. Brodsky Classification

Brodsky classification was used for palatine tonsil size evaluation. The grading is classified as follows: grade 0 = tonsils limited to the bilateral tonsillar fossa; grade 1+ = tonsils occupy less than 25% of the space between the anterior pillars in the oropharynx; grade 2+ = tonsils occupy 25% to 50% of the space between the anterior pillars; grade 3+ = tonsils occupy 50% to 75% of the space between the anterior pillars; grade 4+ = tonsils occupy 75% to 100% of the space between the anterior pillars [8].

#### 2.2.2. Modified Mallampati Score

MMS was used for grading in accordance with tongue size and oral cavity, and higher MMS refers to a larger tongue size. Patients were classified as follows: class I = complete visualization of uvula, tonsils, and palatal arches; class IIa = complete visualization of the uvula while the tonsils and arches are partly hidden; class IIb = visualization of the soft palate and base of the uvula but not tonsils or pillars; class III = visualization of some of the soft palate but not tonsils, pillars, distal soft palate, or base of the uvula; and class IV = only the hard palate is visible [9].

#### 2.2.3. Modified Friedman’s Staging System

Modified Friedman’s staging system is used to predict the success of uvulopalatopharyngoplasty. The system is based on tonsil size, tongue positions, and BMI [10]. The stages are classified as follows: Stage I is defined as patients with tongue position 1 or 2 and tonsil size 3 or 4; and BMI < 40. Stage II is defined as patients with tongue position 1 or 2 and tonsil size 0–2; or tongue position 3 or 4 and tonsil size 3–4; and BMI < 40. Stage III is defined as patients with tongue position 3 or 4, tonsil size 0, 1, or 2; and BMI < 40. Stage IV is defined as patients with tongue position 1, 2, 3, or 4; tonsil size 0, 1, 2, 3, or 4; and BMI > 40 or with skeletal deformities such as micrognathia or mid-face hypoplasia [10]. Lower modified Friedman’s stage means there is a greater chance of success after uvulopalatopharyngoplasty for OSA [14].

#### 2.2.4. Muller’s Test

The patient was evaluated while sitting in an awake condition, and retropalatal and retrolingual obstruction was assessed. All the patients underwent Muller’s test with topical nasal anesthesia and/or vasoconstrictor. An Olympus 3.2 mm fibro-optic scope passed through the nose to survey velum, oropharynx, and tongue base; epiglottis was used for evaluation. Every patient received level dimensional change by inhaling with a blocked nose to cause negative pressure and the collapse percentage of the airway in the velum, oropharynx, and tongue base areas; the epiglottic area was assessed and graded under Wang’s grading system [15]. The collapsibility and dimensional change were recorded as follows: retropalatal anterior and posterior (A–P) collapse grade 0–2; retropalatal lateral collapse grade 0–2; retropalatal circumferential collapse grade 0–2; retroglossal A–P collapse grade 0–2; retoglossal lateral-to-lateral (L–L) collapse grade 0–2; and retroglossal circumferential collapse grade 0–2. Grade 0 indicated no observed airway obstruction, grade 1 indicated partial obstruction, and grade 2 indicated total obstruction [15].

### 2.3. VOTE Classification on DISE

DISE was performed for every patient in the supine position in the operating room, under pulse-oximetry and cardiac rhythm monitoring. One percent lidocaine with topical phenylephrine mixture spray was applied to nares, before an Olympus 3.2 mm fibro-optic scope was passed through the nose to survey velum, oropharynx, tongue base, and epiglottis. During DISE, IV propofol 100 mcg/kg/min was infused by TIVA under TCI-inducing sedation and was maintained by 0.1 to 0.3 mg/kg intermittently for DISE. The sleep depth was evaluated by bispectral index monitoring, in which the level below 70 was considered suitable for DISE records. The VOTE classification was a structure-based evaluation [13] encompassing the degree and configuration of the related obstruction. Four anatomical levels—velum, oropharynx, tongue base, and epiglottis—were assessed for the severity and configuration of obstruction. The velum level of configuration of obstruction was to gain an observation from anteroposterior, lateral, or concentric directions. For oropharynx level, the obstruction was assessed in the lateral direction. The obstruction of the tongue base was observed in the anteroposterior configuration. The anteroposterior and lateral configurations were assessed in an epiglottis level of configuration. The degree of obstruction was classified as 0 (no obstruction), 1 (partial obstruction), and 2 (total obstruction). We clarified the tongue base and oropharynx according to the tonsil tail; above the tonsil tail and below velum we counted it as the oropharynx. Below the tonsil tail and above the vallecular fossa we counted as the tongue base. We diagnosed the site of the upper airway collapse by at least 10 cycles of breathing or snoring during the DISE, from velum to epiglottis. As in the oropharyngeal area, the posterior position collapse was counted as a pro-pharyngeal collapse and anterior collapse by dropping tongue or lingual tonsil hypertrophic-related obstruction is mentioned as a tongue base obstruction. These two regions were part of the same anatomical region in our study.

### 2.4. Statistical Analysis

Statistical analyses were performed using SPSS 19 software package (SPSS Inc., Chicago, USA). Data were expressed by descriptive statistics, and the t-test was used to compare clinical characteristics differences between “not severe” and “severe” OSA patients. The categorical variable of proportion testing was analyzed by chi-square and Fisher’s test. The statistical relationship between different levels of physical examinations (Brodsky classification grade 0 + 1 + 2 vs. grade 3 + 4; MMS class I+II vs. class III+IV; modified Friedman’s staging system stage I vs. stage II+III+IV; Muller’s test grade 0 vs. grade 1 + 2) and DISE (grade 0 or 1 + 2) was analyzed by chi-square test. Spearman’s correlation coefficient was used to analyze the relationships between the physical examinations and related VOTE classification. Cohen’s kappa coefficient (κ) was used to measure inter-rater reliability of different levels of physical examinations and related VOTE classification. Kappa values < 0 were taken as indicating no agreement, 0~0.20 as slight agreement, 0.21~0.40 as fair agreement, 0.41~0.60 as moderate agreement, 0.61~0.80 as substantial agreement, and 0.81~1 as almost-perfect agreement. P-value < 0.05 was considered a statistically significant difference.

## 3. Results

The study sample consisted of 55 patients (44 men and 11 women; age = 44.9 ± 12.9 years, BMI = 30.6 ± 4.7 Kg/m^2^; and AHI = 44.6 ± 22.0/hr). Twenty eight of 55 (50.9%) OSA patients had BMI ≧ 30, and 36 of them (65.5%) had AHI ≧ 30/hr. Comparing the clinical characteristics and physical examinations between the patients with severe OSA (AHI > 30/hr) and not-severe OSA (AHI ≤ 30/hr), there were no significant differences in age, Brodsky classification grade, MMS, modified Friedman’s staging system, Muller’s test and collapse level under DISE (*p* > 0.05) except BMI (*p* < 0.05, Table 1). In the current study, 12 of 55 (21.8%) patients showed one-level collapse and the other 43 of the 55 (78.25%) patients showed multilevel collapse under DISE. Velum was the most common collapse area (90.9%), followed by tongue base, oropharynx, and epiglottis.

The analysis results for correlation coefficients analysis of physical examinations and VOTE during DISE are shown in Table 2. Oropharynx assessment of VOTE during DISE had significantly positive correlations with Brodsky classification and modified Friedman’s staging system (r = 0.69 and r = 0.38 respectively, *p* < 0.05). Tongue assessment of VOTE during DISE also had a significantly positive correlation with modified Friedman’s staging system (r = 0.36, *p* < 0.05). Conversely, no correlation was noted between MMS and tongue assessment of VOTE (*p* > 0.05). VOTE level, retrolingual A–P of Muller’s test and MMS had significant positive correlations (r = 0,26 and r = 0.43 respectively, *p* < 0.05). The VOTE count, Brodsky classification, MMS, modified Friedman’s staging system, and retropalatal L–L of Muller’s test were significantly correlated (*p* < 0.05). 

A kappa statistic was used to further evaluate the different levels of four physical examinations and related VOTE under DISE in Table 3. The concordance between VOTE under DISE, Brodsky classification, or modified Friedman’s staging system was moderate (κ = 0.42, κ = 0.41, respectively). The concordance between VOTE under DISE and MMS was slight (κ = 0.13). There was also a similar slight concordance between VOTE under DISE and Muller’s test (κ = 0.12).

Because the Brodsky classification and oropharynx level in DISE had a moderate concordance in Table 3, the different levels of physical examinations and oropharynx collapse during DISE were reanalyzed. The other sites during Muller’s test (i.e., velum, tongue base, and epiglottis) versus awaking physical examinations are also showed in Table 4. In grades 1 and 2 of oropharynx scoring during DISE, grades 0, 1, and 2 of Brodsky classification were observed in 15 of 41 patients (36.59%). A higher Brodsky classification (grade 3 + 4) was accompanied by oropharynx collapse in 13 of 14 patients (92.86%). A significant relationship was found between Brodsky classification and oropharynx collapse during DISE (*p* < 0.05, Table 4). In grades 1 and 2 of oropharynx scoring under DISE, obstructions at tongue base classified with MMS of I or II were observed in 15 of 27 patients (55.55%). A higher score in MMS (score III + IV) was accompanied by oropharynx collapse in 18 of 28 patients (64.29%). No significant relationship was found between MMS and oropharynx scoring during DISE (*p* > 0.05).

When OSA patients were in stage I of modified Friedman’s staging, oropharynx scoring during DISE was noted in 40% of patients (6/17 patients). A modified Friedman’s staging as stages II, III, and IV was accompanied by oropharynx scoring during DISE in 70% of patients (28/40 patients). Modified Friedman’s staging had a significant relationship with oropharynx collapse during DISE (*p* < 0.05). In grades 1 and 2 of oropharynx scoring during DISE, grade 0 of Muller’s test were noted in 6 of 18 patients (33.33%). A higher Muller’s test (grades 1 and 2) was accompanied by oropharynx collapse in 24 of 37 patients (64.87%). There was a significant relationship between Muller’s test and oropharynx scoring during DISE (*p* < 0.05).

## 4. Discussion

For the OSA patients, a major goal of non-CPAP treatment is the identification of the correct collapse level of the upper airway. We used to evaluate patients with a clinical examination and a grading system, but these methods may show limited value because they are performed while patients are awake. DISE was first described in 1991, providing a similar condition to natural sleep and dynamic assessments [16]. In the present study, indications for DISE are not yet well codified. Kotecha et al. suggested DISE for patients with moderate-to-severe OSAS, in whom CPAP therapy was unacceptable or indeed failed, considering sleep endoscopy as part of further assessment and valuable for targeting treatment [17]. Kezirian et al. recommend DISE after failure of surgery [18]. The aim of this study was to evaluate the relationship between the findings of physical examination and DISE by VOTE. Apart from VOTE, there was another nose oropharynx hypopharynx and larynx (NOHL) grading system for DISE [19], it is not only grading the collapse of oropharyngeal, hypopharynx, and larynx but also the nasal cavity by grade I (0–25%), grade II (25–50%), grade III (50–75%), and grade IV (75–100%). Because VOTE is widely discussed in the literature and our data was also collected by VOTE, we therefore showed all of our study for DISE using the VOTE system; the future physical examination correlation between DISE by NOHL and awaking physical examination is worth doing in the future.

On oropharynx assessment, Fernandez-Julian et al. showed acceptable concordance [20]. M. Blumen et al. also suggest only the tonsils area seems to be accurately assessed on clinical examination [21]. In the current study, we also found a significant relationship between Brodsky classification and oropharynx collapse during DISE. The result is similar to previous studies [20,21]. On tongue base assessment, Aktas et al. revealed no significant relationship between higher MMS and tongue base collapse during DISE [22]. Den Herder et al. also showed no correlation between a large tongue (MMS: III or IV) and collapse level at tongue base during DISE [23]. Our results showed no significant relationship between MMS and tongue base collapse during DISE. Because MMS was used to score a large tongue size, and DISE was used to assess obstruction at tongue base level, both assessments were recorded differently. However, Wang et al. showed a significant correlation between MMS and DISE in the evaluation of tongue base collapse [15]. The different view was that retrolingual obstruction occurred due to muscular hypotonia, resulting in upper airway collapse during sleep.

Fernandez-Julian et al. showed good correlation between DISE and Friedman staging regarding the suggestion of operation [20]. Stage I in modified Friedman’s staging system had higher than an 80% treatment success rate. Stage II~III had a very low treatment success rate and may need to consider multilevel surgery [14]. Soares et al. revealed that patients with Friedman stages II and III had a higher possibility of tongue base obstruction during DISE [24]. Similar to a previous study [20], our results showed that a higher modified Friedman stage (III, IV) was significantly correlated with multilevel collapse. In addition, there was a substantial correlation between physical examination and DISE findings. We could observe a patient’s collapsibility of velum, oropharyngeal, and tongue base in an awake condition. However, perfect matching prediction of upper airway collapse condition still warranted DISE. In addition, there was a new classification written by Friedman in 2017 [10] which added a grading for lingual tonsil hypertrophy. This should be taken into account and might render a higher correlation between DISE and awake clinical physical examinations and so future work is needed. Generally, we should still combine DISE with an awake examination for evaluating the anatomy in order to decrease under diagnosis and to create a proper treatment strategy according to DISE.

Our study aimed to compare the findings of clinical examination with DISE. We found that not all clinical findings showed a significant relationship between DISE findings. A higher score in MMS does not always indicate that obstruction is at the level of the tongue base. Personalizing the level of the obstruction site is essential for correct surgical management in patients with OSA. Certal V.F. et al. showed that surgical treatment changed after DISE in 50.24% cases [25]. In conclusion, DISE may give us a more precise evaluation of obstruction level, especially on the level of the tongue base. Currently, there is no study showing a better treatment success rate in DISE-oriented therapy compared to non-DISE-oriented therapy. However, selection bias was introduced by collecting more patients with deviated nasal septum in the non-DISE group. The treatment methods differ among tertiary referral centers [26].

The physical examination is considered to have correlation with DISE because both are used to evaluate the upper airway. However, in our study, the highest correlation coefficient was 0.69, between Brodsky classification and oropharynx, which represented a moderate correlation, while the others showed a weak-to-low correlation. Muller’s test was also used to evaluate the correlation with DISE. We found that the site of collapse determined at retropalatal level with L–L direction had a weak correlation with VOTE count and the site of collapse determined at the retrolingual level with an A–P direction had a weak correlation with VOTE level. Therefore, in clinical practice, we should not just perform a physical examination but should also arrange DISE. In a North American DISE-oriented sleep surgery outcome study, the conclusion was offered that surgical outcomes were not correlated with velum and epiglottis-related obstruction or the degree of epiglottis-related obstruction, but were instead associated with tonsil size and BMI. They still consider DISE to be an important tool for evaluating collapse at tongue base and oropharyngeal lateral walls and an important diagnostic tool toward making proper sleep surgery plans for each OSA patient [27]. 

There are several limitations in our study. First, the case number of this study was limited. Second, we use propofol under bispectral index monitoring for DISE. Generally, propofol has a quicker onset, a shorter half-life, and can demonstrate larger degrees of obstruction, which may reflect more accurately what happens during rapid-eye-movement sleep [28]. However, there was no gold standard for sedative choice. Third, the findings were based on VOTE but not on the NOHL [19] system during DISE. The VOTE is a subjective examination and future study of NOHL grading for DISE and interrater reliability studies is warranted. Fourth, there were possible clinical finding differences from different observes, however, the same senior surgeon with the same senior sleep fellow did all the clinical findings repeatedly to reduce the grading errors as much as possible. Fifth, velum is the most common collapse area (90.9%), but there is no standard method for evaluation. Although Woodson BT et al. provide a method for the scoring of individual landmarks for the velopharynx [29], related research is limited.

## 5. Conclusions

The study revealed that physical examinations, i.e., Brodsky classification and Friedman staging, have a significant relationship with DISE, but the level of tongue base is uncertain between DISE and MMS. DISE can be a reliable method for evaluating upper airway dynamics during sleep. DISE, with all its limitations and the relevance of its findings so far, goes beyond the objective of simply identifying collapse with precision, but rather helps in understanding the collapse of the identified sites.

## Figures and Tables

**Table 1:** Clinical characteristics between patients with not-severe and severe OSA.

	Not Severe AHI < 30	Severe AHI ≧ 30	*p* Value
Age (years)	46.01 ± 10.23	47.05 ± 11.19	0.89
BMI(kg/m^2^)	27.32 ± 3.11	31.15 ± 4.28	0.04 *
Brodsky classification grade	2.24 ± 0.83	3.45 ± 0.56	0.06
MMS	3.14 ± 0.95	2.75 ± 0.98	0.12
Modified Friedman’s staging system	2.86 ± 1.35	3.98 ± 0.86	0.05
Muller’s test	0.37 ± 0.74	0.51 ± 0.76	0.08
VOTE sites	2.11 ± 1.43	2.31 ± 1.55	0.40

* *p* < 0.05. AHI, apnea-hypopnea index; BMI, body mass index; MMS, modified Mallampati score; VOTE, Velum, Oropharynx, Tongue base, and Epiglottis classification.

**Table 2:** Pearson correlation coefficients between physical examination and related VOTE under DISE.

Items	Velum	Oropharynx	Tongue	Epiglottis	VOTE Level	VOTE Count
Brodsky classification	-	0.69	-	-	0.15	−0.26
*p*-value		0.001 *			0.24	0.04 *
MMS	-	-	0.23	-	0.43	0.28
*p*-value			0.08		0.001 *	0.03 *
Modified Friedman’s staging system	-	0.38	0.36	-	0.13	0.30
*p*-value		0.003 *	0.01 *		0.31	0.02 *
Muller’s test						
Retropalatal A–P (velum)	−0.14	0.11	−0.06	0.09	0.11	−0.16
*p*-value	0.27	0.40	0.65	0.49	0.41	0.24
Retropalatal L–L (velum)	0.18	0.02	0.08	0.26	0.04	0.26
*p*-value	0.16	0.83	0.52	0.05	0.73	0.04 *
Retropalatal-C (velum)	−0.05	0.13	−0.05	−0.19	0.17	−0.13
*p*-value	0.70	0.32	0.66	0.14	0.20	0.34
Retrolingual A–P (tongue)	0.001	0.02	0.02	−0.07	0.26	−0.03
*p-* value	1.00	0.87	0.83	0.60	0.04 *	0.81
Retrolingual C (tongue)	0.21	−0.22	0.10	−0.11	−0.19	0.12
*p-* value	0.11	0.10	0.42	0.38	0.14	0.38
Retrolingual L–L (oropharynx)	−0.13	0.17	0.06	0.22	0.21	−0.007
*p-* value	0.32	0.19	0.66	0.10	0.11	0.95

* *p* < 0.05. VOTE, Velum, Oropharynx, Tongue base, and Epiglottis classification, MMS, modified Mallampati score; A–P, collapse in anterior–posterior direction; L–L, lateral-to-lateral diection; C, circumferential collapse. VOTE Count is a summation of collapse degrees of VOTE levels.

**Table 3:** Correlation coefficients between different levels of Brodsky classification, MMS, Friedman stage and different level DISE.

	DISE										
	Oropharynx			Tongue Base			O+T			V+O+T+E	
Assessment 1	0	≥1	Assessment 2	0	≥1	Assessment 3	1	>1	Assessment 4	1	>1
Brodsky classification			MMS			Modified Friedman’s staging system			Muller’s test		
Grade 0 + 1 + 2	26	15	Class I+II	13	15	Stage I	10	5	Grade 0	10	8
Grade 3 + 4	1	13	Class III+IV	9	18	Stage II+III+IV	9	31	Grade 1 + 2	16	21
Kappa (95% CI)	0.42 (0.21–0.62)			0.13 (−0.12–0.38)			0.41 (0.20–0.65)			0.12 (0.10–0.43)	

MMS, modified Mallampati score; DISE, drug-induced sleep endoscopy; VOTE, Velum(V), Oropharynx(O), Tongue base(T), and Epiglottis(E).

**Table 4:** Comparison of different levels of physical examinations and oropharynx collapse during DISE.

	Oropharynx Scoring during DISE		
	Grade 0	Grade 1 + 2	*p* Value
Brodsky classification			
Grade 0 + 1 + 2 (n = 41)	26 (63.41%)	15 (36.59%)	0.001 *
Grade 3 + 4 (n = 14)	1 (7.14%)	13 (92.86%)	
MMS			
Class I + II (n = 27)	12 (44.44%)	15 (55.55%)	0.51
Class III + IV (n = 28)	10 (35.71%)	18 (64.29%)	
Modified Friedman’s staging system			
Stage I (n = 15)	9 (60%)	6 (40%)	0.04 *
Stage II + III + IV (n = 40)	12 (30%)	28 (70%)	
Muller’s test			
Grade 0 (n = 18)	12 (55.56%)	6 (44.44%)	0.02 *
Grade 1 + 2 (n = 37)	13 (35.13%)	24 (64.87%)	

* *p* < 0.05 by Chi-square test for correlation between groups. MMS, modified Mallampati score; DISE, drug-induced sleep endoscopy.

## Abbreviations

AHI	Apnea-hypopnea index
BMI	Body mass index
CPAP	continuous positive airway pressure
DISE	drug-induced sleep endoscopy
NOHL	nose oropharynx hypopharynx and larynx
OSA	obstructive sleep apnea
MMS	modified Mallampati score
PSG	polysomnography
TCI	target control infusion
TIVA	total intravenous anesthesia
VOTE	Velum, Oropharynx, Tongue base, and Epiglottis
